# Supplementary material for: MONET: The Minor Body Generator Tool at DART Lab
Source: Sensors (Basel). 2024 Jun 5;24(11):3658. doi: 10.3390/s24113658 (PMC11175308; doi:10.3390/s24113658)
Supplement: Supplementary file 1 [file sensors-24-03658-s001.zip › sensors-2974505-supplementary.pdf]

Supplementary Material

In this appendix, the Blender node trees for the implementation of surface morphological features are presented for reproducibility purposes. Figure S1 illustrates the overall material node tree, whose outputs serve as inputs for both the reflectivity model and the output material. Within this tree, various groups are incorporated to achieve a realistic appearance for the minor body, introducing features such as craters and surface roughness. Specifically, Figure S2 displays the content of the roughness node tree, while Figures S3 and S4 contribute to the generation of craters. Figure S3 is essential to avoid perfectly circular craters. As already mentioned in Section 2.3.2, Figure S4 highlights that larger craters do not interact with the roughness group, resulting in a rough appearance in their inner part. It is important to note that these node trees are employed for the default minor body families. When users opt for using their own parameters, the rubble-pile node tree is employed, with adjustments made to its parameters.

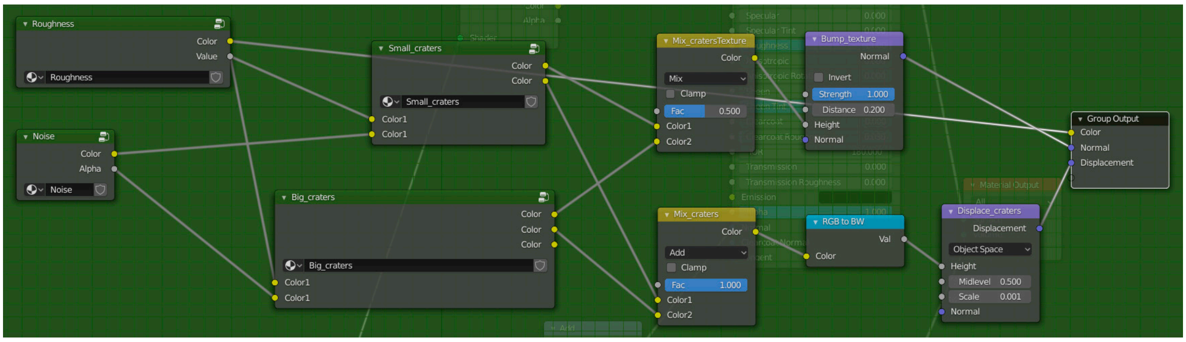

Figure S1: Blender material node tree with all the groups composing the tree, namely, *roughness*, *noise*, *small craters*, and *big craters*.

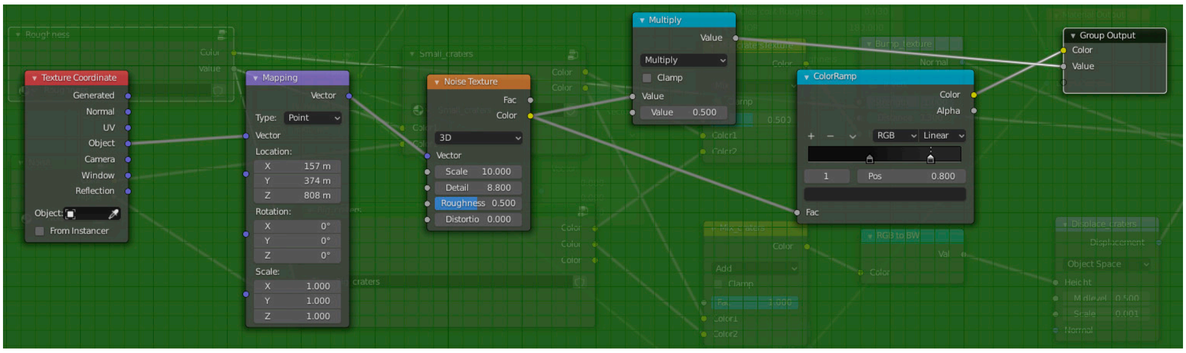

(a)

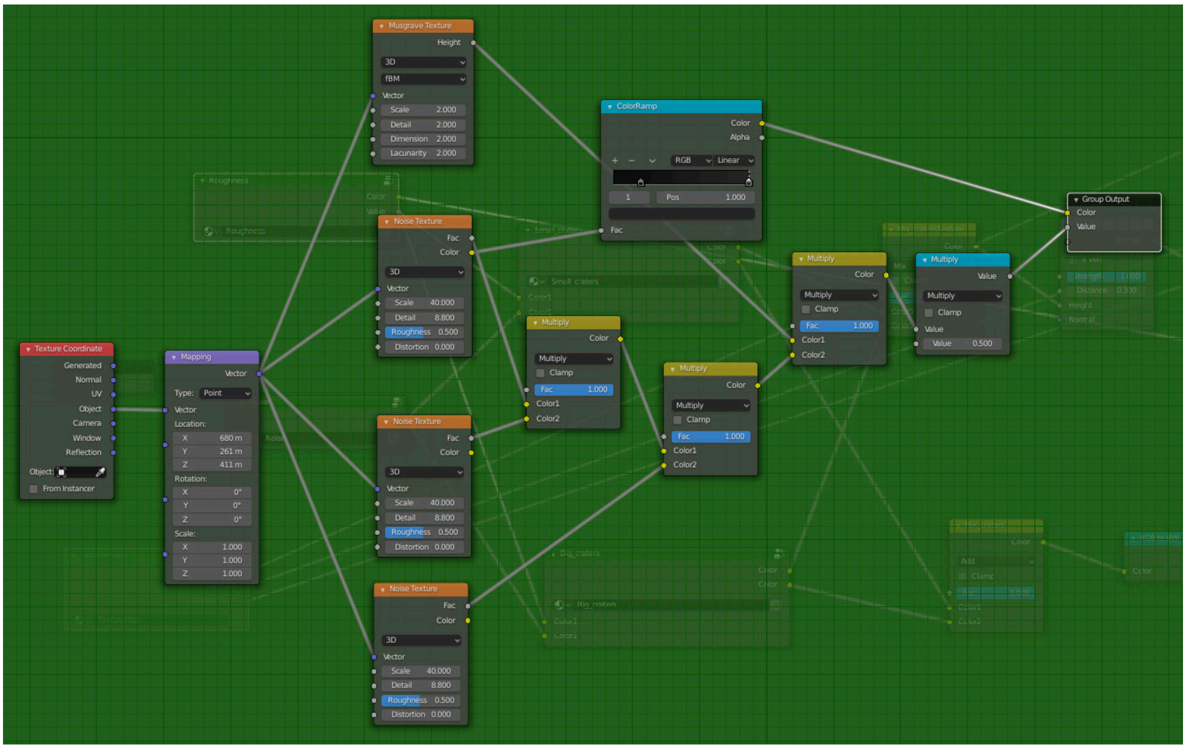

(b)

Figure S2: *Roughness* group node trees for (a) rubble-pile and (b) comet-like bodies.

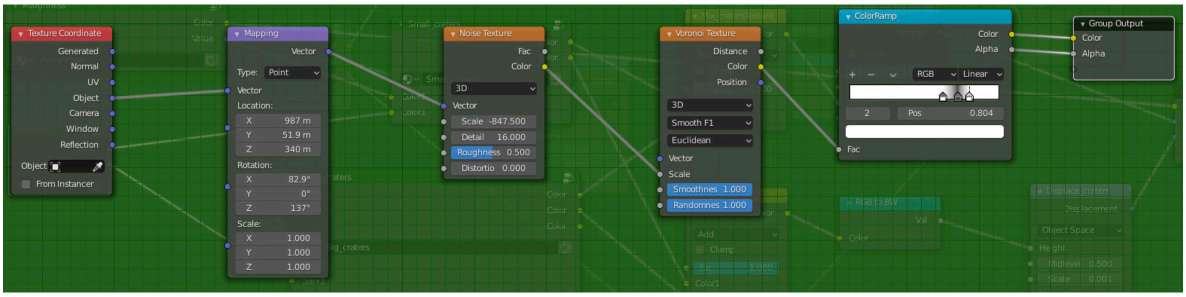

Figure S3: *Noise* group node tree.

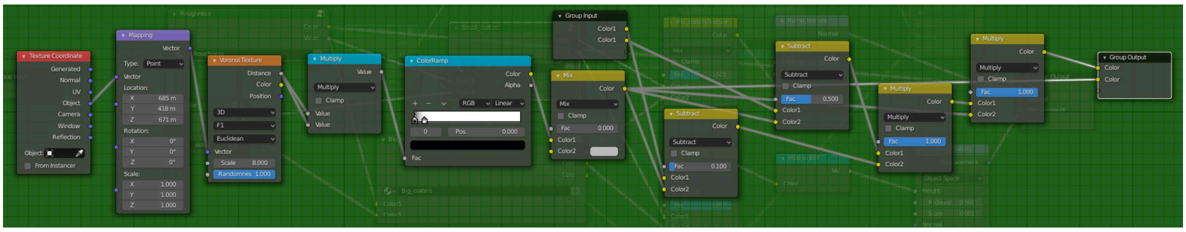

(a)

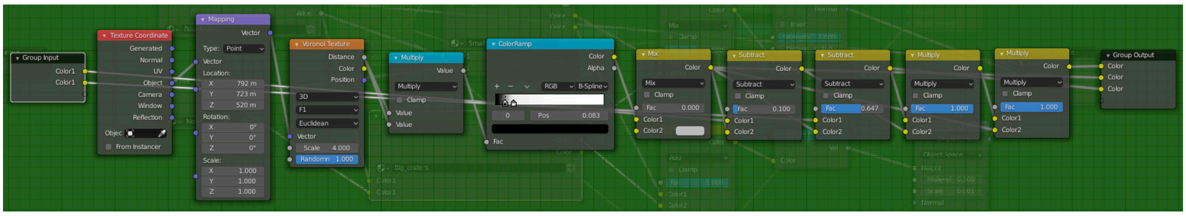

(b)

Figure S4: Craters group node trees for (a) *small craters* and (b) *big craters*.
